# Scientific conclusions need not be accurate, justified, or believed by their authors

**DOI:** 10.1007/s11229-021-03158-9

**Published:** 2021-04-21

**Authors:** Haixin Dang, Liam Kofi Bright

**Affiliations:** 1 The School of Philosophy, Religion and History of Science, Botany House, University of Leeds, Leeds, LS2 9JT UK; 2 Philosophy, Logic and Scientific Method, Lakatos Building, London School of Economics, Houghton Street, London, WC2A 2AE UK

**Keywords:** Social epistemology of science, Norms of assertion, Science communication

## Abstract

We argue that the main results of scientific papers may appropriately be published even if they are false, unjustified, and not believed to be true or justified by their author. To defend this claim we draw upon the literature studying the norms of assertion, and consider how they would apply if one attempted to hold claims made in scientific papers to their strictures, as assertions and discovery claims in scientific papers seem naturally analogous. We first use a case study of William H. Bragg’s early twentieth century work in physics to demonstrate that successful science has in fact violated these norms. We then argue that features of the social epistemic arrangement of science which are necessary for its long run success require that we do not hold claims of scientific results to their standards. We end by making a suggestion about the norms that it would be appropriate to hold scientific claims to, along with an explanation of why the social epistemology of science—considered as an instance of collective inquiry—would require such apparently lax norms for claims to be put forward.

## Introduction

We hope that by inquiring together we may eventually discover the truth. Or, failing that, at least to achieve some other epistemic aim—we shall assume it is truth from here on out, though nothing turns on that choice of aim in particular. Whatever it may be, to achieve our long run epistemic goals, individuals or small teams must periodically make local contributions. The field of biology, say, has succeeded in its aim if it discovers the truth about biological systems, but what does this entail for how we evaluate individual papers published in biology journals? Should we say each of those must discover the truth about its particular topic matter? In general, if we are to make progress in collective inquiry, what do we require from the individual contributors?

Even if we grant that the relationship between our expectations for the whole process and our expectations for individual inputs must be complex (see Mayo-Wilson et al., 2011), we evidently hold such individual contributions to some standards. The process of peer review as it now exists (on which see Heesen & Bright, 2020) seems to presuppose that there are conditions under which it would be inappropriate to bring a conclusion to the collective attention of one’s field. Likewise, the very fact that many consider widespread replication failure (see e.g. Open Science Collaboration, 2015) to be problematic suggests that they hoped for more from the principal claims of those fields in which that debate is happening. So what exactly are those norms which, in the good case, a particular local piece of scientific inquiry will satisfy in putting a conclusion forward?

To answer this question, we turn for inspiration to a branch of analytic epistemology and philosophy of language that concerns itself with the conditions under which particular claims are properly put forward. We will understand “properly put forward” here to mean uttered in a way that coheres with the contextually appropriate utterance norms. It is often claimed that assertions are utterances held to certain norms, called norms of assertion. This does not mean that in our assertions we always (or even usually) meet the appropriate norms. But rather that assertions qua assertions are improper if they fail to satisfy the requirements set by these norms—indeed in some cases it is felt that it is constitutive of something being an assertion that it is held to these norms.

In this paper, as a way into the general question of how individual pieces of inquiry may contribute to a successful joint endeavour, we explore whether the utterances made by scientists when announcing their findings and results are held to any of these clusters of norms of assertion. The analogy between assertions and discovery claims in scientific papers seems natural—intuitively, they are both ways of putting forward a claim about how the world actually is. So as a starting place for inquiry, it seems a good place to begin in thinking about what sort of norms one should satisfy in putting a claim forward. Take, for instance, a discovery claim drawn from the abstract from (at time of writing) a paper in the latest issue of the journal *Cell*. The authors claim that their results “demonstrate'' that “loss of Cx3cr1 in CNS-myeloid triggers a Cxcl10-mediated vicious cycle, cultivating a br-met-promoting, immune-suppressive niche” (Guldner et al 2020). On its face, to claim to have demonstrated something is to claim that it has been shown to be true and what is being claimed true constitutes an assertion about how the immune system in the brain operates. However, we will find that such claims made about individual scientific contributions ought not be held to typical norms of assertion. We do not take a stand on whether the scientific conclusions should be held to be assertions or not, but we do claim that reflection upon why they are not held to typical norms of assertion is illuminating as to the general character of collective inquiry and the relationship its individual subcomponents must bear to the overall endeavour.

There are three clusters of norms in the norms of assertion literature. The first cluster of norms studied by analytic epistemologists are the factive norms. Most famously there is the knowledge norm, owed to Timothy Williamson. According to this norm "[o]ne must: assert p only if one knows p" (Williamson, 2000: 243). In a similar vein García-Carpintero (2004) proposes the norm that one must assert p only if one's audience comes thereby to be in a position to know that p (156). Matthew Weiner, on the other hand, (2005), proposed a truth norm: assert only what is true. Jason Stanley has raised the suggestion that one may assert that p only if one knows that p (or is in a position to know that p) on the basis of evidence that gives one the highest degree of justification for one's belief that p. (2008: 35). MacFarlane (2014: 108) proposes a norm that one ought to retract an (unretracted) assertion if it turns out not to be true. This first class of proposals is united in holding assertions improper if they are not true.

The second cluster are justification norms. For instance, Jennifer Lackey, reflecting on what she calls "self-less assertions" in which one properly asserts what one does not believe, proposes that assertions are held to the norm "that it is reasonable to believe that p, and that the speaker asserts that p for this reason (even if in fact not believing that p)" (quotes from Pagin, 2016; summarising Lackey, 2007). Gerken (2015a), reflecting specifically on lab mates or collaborating scientists discussing ongoing research, advocates (in that context) a norm wherein scientists must be “discursively justified”—justified in their claims, and able to explain that justification to any who request reasons. Kvanvig (2009: 145, 2011: 249–250) has proposed that the norm of assertion is that one needs to be justified if one asserts, and that the relevant concept of justification is such that it is sufficient for knowledge, provided the proposition is both believed and true (and the justification is undefeated).

The third cluster are belief norms. Taking a cue from Grice (1961), Bach (2008) argues that the only relevant rule on assertion is belief: “In the case of assertion, to be sincere is to hold the belief one expresses. So we can say that belief, not knowledge, is the rule on assertion” (77). Others have argued that it is only proper to assert what one believes justifiedly. Douven (2006) defends the view that one should only assert what one believes to be rationally credible.

We ground our inquiry in an exploration of whether norms of any of these types should be applied to scientific claims, and if not, why not. The aim of this paper is not to entirely dismiss the proposed norms of assertion as they are currently studied by analytic epistemologists. Rather, our aim is to demonstrate that these norms of assertion are not appropriate for important classes of scientific claims. In the next section we will say more about the sorts of claims we wish to study, and give a simple abstract example of such claims being made. We then argue that the points illustrated in this abstract case can also be studied in a more detailed historical case, and that we can generalise our lessons to modern scientific practice. We will give two different theoretical arguments for this: an argument from division of cognitive labour and an argument from the pessimistic meta-induction.

## Public avowals in science

To be more precise about the target of this paper, we will call the class of utterances that we are interested in “public avowals.” These are utterances made by scientists aimed at informing the wider scientific community of some results obtained: conclusions in papers in peer-reviewed journals, conference presentations, posters, online pre-prints, etc. Indeed, these are the kind of utterances which we most commonly associate with the scientific community. Open up an issue of *Science*, for example, and one may read that “early vertebrate clades, both jawed and jawless, originated in restricted, shallow intertidal-subtidal environments” (Sallan et al., 2018). Public avowals may also be much more speculative, hesitant, or demure, such as this highly qualified statement appearing in the abstract of a recent paper in the *American Journal of Sociology*: “[a]pparent changes in disparities across census tracts may result partly from a higher level of sampling variation and bias due to the smaller sample” (Logan et al., 2018).

We do not mean by public avowals, therefore, definitive statements about the world that are confidently maintained. Instead, we simply mean claims that scientists feel in a position to put forward as a conclusion of their research. The conclusion claims of scientific papers are sometimes put forward in a qualified or hedged manner, but they are still statements that publicly report the results of research. Thinking about the norms or standards these public avowals are or ought to be held to is informative in telling us how to think about the central contributions of novel scientific papers.

Gerken (2015b) has argued that testimony between collaborating scientists during the process of collaboration are held to special epistemic norms. He argues that such *intra-scientific* testimony ought to be held to the standard of discursive justification. The kinds of utterances we are concerned with differ from Gerken. We are interested in the norms that govern *inter-scientific* testimony: claims made by scientists or collaborations aimed at the wider scientific community. These types of claims are meant to convey the products of scientific inquiry, rather than made during the course of that inquiry, and are made in public venues where the primary audience is other scientists. The paradigmatic cases of inter-scientific testimony are scientific articles published in specialist journals aimed at other expert scientists (for norms concerning co-authoring scientific papers see Huebner et al., 2017; Bright et al., 2018). This is because our focus is on the question of how individual contributions are made to a larger project of scientific inquiry; it is hence the relationship between an individual scientist and her broader scientific discipline that most concerns us.

Public avowals in science should not be confused with *extra-scientific* testimony or “public scientific testimony.” Statements from scientists aimed at policy-makers are not the type of utterances we are interested in. It is important to distinguish between claims aimed at the scientific community and claims aimed at the general public or policy-makers. The IPCC assessment report on climate change, for example, while made publicly, is primarily aimed at political bodies, and such testimony is properly held to a different standard. Extra-scientific testimony is hence not the target of our paper.

It is very important that we recognize that “public scientific avowals” are held to different epistemic norms than that of “public scientific testimony.” Confusing “public scientific avowals” and “public scientific testimony” can lead to disastrous misunderstandings about science communication. To anticipate some results of our argument, if a layperson were to only follow the specialist journals, the layperson may come to the conclusion that scientists often report false things, after reading many contradictory avowals made at the conclusion of competing cutting-edge research papers. However, it would be a mistake for the layperson to think that when scientists make reports to policy-makers that these reports also have the same epistemic status as papers in scientific journals written for a primarily specialist audience.

We will now argue that public scientific avowals should not be held to factive norms, justification norms, and belief norms. That is to say, we will argue that public avowals which violate all such norms are entirely appropriate in scientific inquiry. Our argument depends on how scientific findings are actually communicated in the scientific community, or the role such communications play. To illustrate the role of public scientific avowals in science, consider this scenario which a scientist may often find herself in:

Zahra is a scientist working at the cutting edge of her field. Based on her research, she comes up with a new hypothesis. She diligently pursues inquiry according to the best practices of her field for many months. Her new hypothesis would be considered an important breakthrough discovery. Zahra knows that many more studies will have to be done in the future in order to confirm her hypothesis. Further, she has read the current literature and realizes that the existing research in her field does not, on net, support her hypothesis. She does not believe that she has conclusively proven the new hypothesis. Nonetheless, Zahra sends a paper reporting her hypothesis to the leading journal in her subdiscipline. In the abstract of the paper, the conclusion, and talks she gives on her work, she advocates for her hypothesis. Peer reviewers, while also sceptical of the new hypothesis, believed that her research had been carried out according to best known practices and her paper would be a valuable contribution to the field. Her paper, which purports to have advanced a new hypothesis, is published and widely read by members of her community. In subsequent years, additional research in her field conclusively demonstrates that Zahra’s hypothesis was false.

Public scientific avowals like Zahra’s maintaining her hypothesis do not live up to the standards set by the norms of assertion. Zahra’s avowals were false. Her avowals were not justified by the total evidence available to her, since she is acquainted with the existing research in her field which does not support her hypothesis. Furthermore, Zahra herself did not fully believe in her avowals. Nonetheless, we believe that some such avowals that fail norms we hold assertions to can still be important to the epistemic success of science. In fact, Zahra’s conduct is exactly how scientists ought to act in order to successfully communicate scientific findings. During active scientific research, public scientific avowals will often fail to meet the norms of assertion, yet scientists still need to continue to make avowals which report their findings to other members of their community.

How widespread and generalizable are public scientific avowals that fail to meet the norms of assertion? Next, we present a short case study from the history of physics, which further illustrates how public avowals actually function among scientists. Scientists do sometimes report false, unknown and unjustified things that they do not believe, and this is an acceptable part of the scientific process.

## Case study: scientific avowals during periods of active inquiry

In the early twentieth century, physicists were discovering a variety of new physical phenomena which were known as radioactivity. There was intense research into the true nature of the mysterious products that were emitted from cathode ray tubes: were they particles or waves? Of the cathode ray products, X-rays and γ-rays were particularly perplexing. It was immediately discovered that X-rays reacted with photographic plates, suggesting that they were a form of light, which would hence suggest they are wave phenomena. However, they also reacted with other materials like particles. On the other hand, γ-rays emissions were almost always accompanied with α and β-particles. It was an open question whether γ-rays and X-rays were material—that is, made up of particles—or whether they were waves in the electromagnetic aether. Investigations into the perplexing properties of all these forms of radioactivity were a major research program in the early twentieth century. Many different theories were proposed to account for the conflicting experimental data.

An early researcher in radioactivity, William Henry Bragg proposed a neutral material particle theory of γ and X-rays, which he defended in a series of scientific articles from 1907 to 1912 (Bragg, 1907, 1908a, 1908b, 1908c, 1910, 1911, 1912). According to this material theory, γ and X-rays were in fact made up of positively charged α-particle and a negatively charged β-particle bound together, making a *neutral pair*. Bragg argued that the leading theory at the time, the aether pulse theory (a form of wave theory), could not fully explain his experimental results regarding γ and X-rays. An aether pulse was thought of like a disturbance or ripple in the electromagnetic aether. Bragg published a paper in 1907 in the leading physics journal of the day, first criticizing the available evidence of both γ and X-rays as being an aether pulse, and also advancing his own material theory of neutral pairs:

It appears, therefore, that all the known properties of the γ rays are satisfied on the hypothesis that they consist of neutral pairs. (Bragg, 1907, 441)

To sum up, it is clear that a stream of X rays contains some æther pulses, but it is not easy to explain all the properties of X rays on the æther-pulse theory. The explanations are easier if the rays are supposed to consist mainly of neutral pairs; and the existence of such pairs is not improbable a priori. (Bragg, 1907, 448).

Bragg made clear public avowals to the effect that both γ and X-rays had been shown to be material, such as in a 1908 letter to *Nature*:

Meanwhile, I will point out that the experimental proof of the material nature of the γ rays carries with it, almost surely, a corresponding proof as regards the X-rays. (Bragg, 1908a, 270)

But when pressed on the virtues of the competing aether pulse theory by fellow physicists, Bragg follows up in another 1908 letter to *Nature*:

If I admit the existence of ether pulse, I do not thereby weaken my contention that the most important and effective part of γ and X ray radiation is material. (Bragg, 1908b, 560)

In these series of articles and letters to *Nature* (see also Bragg, 1908c), Bragg is putting forward to the scientific community his theory that both γ and X-rays are material particles. His avowals here are not definitive, as Bragg often presents his hypothesis as a conditional or qualifies his statements with hedges like “almost surely.” Nonetheless, over the course of several years, Bragg published extensively defending his hypothesis against alternatives, pointing out virtues of his view over others, and was clearly putting forward his hypothesis as the conclusion to be drawn from his experiments on γ and X-rays.

Bragg’s public avowal of the neutral material particle theory did not satisfy the norms associated with assertion. First, the neutral material particle theory was false. γ and X-rays are not material particles. γ and X-rays are not made of neutral pairs, consisting of negatively and positively charged particles bound together. Even in 1907, Bragg theory was viewed as being implausible by scientists in the physics community (Wheaton, 1983, 94). A few years later, in 1912, a decisive experiment was conducted by diffracting X-rays through crystals that settled the matter altogether. X-rays cannot be made up of material particles if they can be diffracted; X-rays must be waves. Today, physicists believe that both γ and X-rays are forms of light, composed of photons that exhibit wave-particle duality. There is no aether.

Furthermore, Bragg’s public avowals were not justified on the total evidence, on balance, available at the time. In his early 1907 paper Bragg knew full well that his theory went against the available evidence at the time. Previous scattering experiments from Charles Barkla in 1905 have been taken as corroborating evidence for the pulse theory. He knew he was opposing a "widely accepted" and "ably advocated" theory of γ and X-rays as aether pulses (Wheaton, 1981, 1983). According to Bruce Wheaton, one of the leading historians on this historical episode, the total evidence, on balance, was not on Bragg’s side:

While Bragg had been developing his novel hypothesis, Barkla's experiments had turned up new and remarkably homogeneous secondary x rays. In its fully interpreted form, this evidence would prove to be virtually unanswerable by Bragg. (Wheaton, 1983: 90)

Bragg was well acquainted with Barkla’s criticisms and corresponded with him about his experimental results throughout this period—they in fact exchanged a long chain of public letters in *Nature* at this time. Barkla’s results on both scattering and secondary X-rays cannot be accounted for on Bragg’s theory. So Bragg’s avowals cannot be justified on the total evidence available to him.

Finally, Bragg did not believe in his theory. This is more apparent in Bragg’s personal letters. In 1910, Bragg had been corresponding with Arnold Sommerfeld, a leading German physicist. While Bragg had continued to publicly defend his particle theory, in private, Bragg was more candid about how he considered his theory:

I am very far from being averse to a reconcilement of a corpuscular and a wave theory: I think that some day it must come… I have suggested the neutral pair form myself: but I do not wish to press this unduly or be dogmatic about it. It seems to me to be the best model to be devised at present, and I have no right to claim more. (reproduced in Wheaton, 1981: 272)

Here, Bragg appears to admit that he is only committed to the neutral pair theory as a “suggestion” or the “best model at present.” Dogmatic belief in the neutral pair theory was untenable because shortly after Bragg’s first proposal of the theory, the α-particle was ruled out as a possible candidate for the positive component of the neutral pair. Bragg had to defend the possibility of an unknown positive particle as well as the neutral pair itself. The neutral pair theory was difficult to defend in face of mounting evidence against material theories, which is why Bragg was so circumspect about his doxastic state in his private letters.

While Bragg’s public avowals about the neutral pair theory of γ and X-rays were false, unjustified on total evidence, and were not believed, we want to maintain that these avowals did not violate the norms of inter-scientific communication. Bragg, after all, was awarded the Nobel Prize in 1915 for his work on X-ray crystallography, a new field born out of his early work on the nature of X-rays. Furthermore, Bragg’s X-ray publications were widely read and discussed by other physicists and spurred decades of important research around X-rays. Bragg’s biggest critic, Charles Barkla, would later win the Nobel Prize in 1917 also for his own work on X-rays scattering, which were in part driven by Bragg’s provocative writings.

Let us summarise, then, what this historical episode demonstrates. In a series of scientific public avowals Bragg defended a theory, the neutral material particle hypothesis. His conduct over the course of this research was felt to be sufficiently meritorious that it did not interfere with him winning the highest honour a scientist may achieve—the false theory spurred valuable research into the diffraction patterns of X-rays. Throughout this period, however, many of the avowals he made were false; by the time the Nobel was awarded this was certainly known to the physics community. As Bragg himself admits the full weight of evidence was not clearly on his side, and for this reason he was throughout privately quite circumspect about the extent to which he even believed his claims about the neutral pair. His public avowals, therefore, were neither true, nor justified by the total evidence available, nor believed to be either true or justified by Bragg himself. In the coming sections we shall argue that far from being unusual or evidence of some epistemic defect, this is a proper state for scientific public avowals.

## Norms for public avowals

The proper social epistemic functioning of science requires that public avowals in science fail to satisfy the norms we surveyed at the beginning. We take it to be uncontroversial that if there will be sustained and organised inquiry, any collective inquiry of the sort that science in fact is, there must be successful communication of the latest results and ideas about them. We will assume that the norms governing utterances communicating those results and ideas must reflect and endorse this state of affairs. Any norm for making avowals which rendered it impermissible to put forward just those claims which constitute necessary features of collective inquiry would be misaligned with the purpose of the activity it is supposed to govern. The scientific community has developed norms, both explicit and implicit, which govern utterances that are appropriate for communicating scientific findings to other scientists. We shall argue that these do not look like any of the purported norms of assertion, and for good reason.

First, note the role of the division of cognitive labour. It is well recognised in philosophy of science that such a division is an important strategy for collective inquiry (Kitcher, 1990; Strevens, 2006). Scientists invest time and resources on different approaches to a research domain. For example, during early investigations into γ and X-ray behavior, different researchers used different techniques to study γ and X-ray behavior. This division of labor is often not explicitly planned; rather the limited resources, credit and incentive structures of science encourage scientists to pursue new avenues of research and use different methodologies (see Kummerfeld & Zollman, 2015; Zollman, 2018).

This division of cognitive labor means that during periods of active inquiry, scientists will often be publishing discoveries which are seemingly in conflict with each other. This in fact occurred during the γ and X-ray episode. Bragg investigated the particle-wave problem using, what we call today, hard X-rays, which are higher in energy. Charles Barkla (Bragg’s leading critic and fellow Nobel laureate) investigated using, what we call today, soft X-rays, which are lower in energy. This is because Bragg and Barkla used slightly different techniques for producing X-rays, due to the limited availability of radioactive source materials. They also used different experimental techniques to look at X-ray behavior (see Wheaton, 1983). This led them to come to radically different conclusions about the nature of X-rays. Bragg’s experiments seemed to show that hard X-rays behaved like particles, because he observed large transfers of energies which is indicative of a particle hitting others. Since γ rays are even higher in energy than X-rays, Bragg was able to observe particle-like behaviors. Barkla, on the other hand, observed his soft X-rays to behave more consistently like waves.

At most one of these models of X-rays could have been correct (as it turned out neither are correct), yet both scientists had to publicise their work if scientific progress were to be made. Public avowals which break factive norms are thus epistemically beneficial and ought to be encouraged. Without them we would have never found out eventually that how X-rays are produced changes the energy of the X-rays—this was a fact we only discovered because Bragg and Barkla came to different results due to how they were generating X-rays in their labs.

Moving beyond the necessity of the division of labour, the case study can be placed in the context of a broader historical argument. This builds on the kind of observations that inspired the pessimistic meta-induction (which typically focused on physics) and Stanford’s (2006) problem of unconceived alternatives (which focused on the history of biology). This can be supplemented by recent work on replicability in psychology (Open Science Collaboration, 2015) and cancer research (Begley & Ellis, 2012). Frequent false public avowals are a necessary part of scientific progress, especially in areas of active inquiry. While scientific realists have argued that the history of science is also full of epistemic successes (for example, see Fahrbach, 2011, 2017), it is nevertheless the case that individual contributions to science in areas of active inquiry are prone to error and are continuously replaced by new errors.

These historical arguments can be supplemented with theoretical arguments. All our inquiry inevitably takes place in a situation of scarce resources and competing demands on our attention. Where this is the case, we have theoretical reason to believe that scientists will not gather as much data as a disinterested epistemic planner would have them gather (Heesen, 2015, 2018). Further, some of our inquiry takes place in contexts where there are weak signals, sparse data, and considerable difficulty in running replications. When this is so, limitations to what is possible through statistical inference give us reason to suppose that most of our published findings will be false (Ioannidis, 2005; Romero, 2016).

These facts together suggest we have not found a better way of communicating findings than one in which there are frequent false avowals. As the X-ray case shows, it is crucial for false theories to be put forward, so to encourage more research; Bragg’s neutral pair theory of X-ray was directly responsible for sparking interest in studying radioactivity among many other physicists. If we agree with versions of the pessimistic meta-induction wherein new scientific theories tend to demonstrate pervasive reference failure in their previous iterations, it means that almost all scientific public avowals turn out to be false.

What does the necessity of dividing labour, and the pessimistic conclusions about present and past science, tell us about our norms for scientific public avowals? We think they quickly rule out factive norms. For factive norms would judge inappropriate a great part of those avowals that are necessary for the progress of any collective inquiry which must divide labour and proceed by correcting errors. It is not just that we in fact often will say false things in the course of inquiry, but rather that inquiry could not proceed in a way that was even remotely successful if we did not do so. Given the ubiquity and necessity of such things to collective inquiry this renders factive norms simply inappropriate as a means of deciding what conclusions ought to be put forward from individual contributions to the wider project. The norms of inquiry should not rule out necessary parts of the process.

In fact, we note, there have been many who have argued that factive norms would not even be appropriate for reporting the results of “completed science”, whatever that might be. Consideration of the nature of physical laws (Cartwright, 1983), scientific models (Frigg & Nguyen, 2016), or what it would be to achieve understanding through science (Dellsén, 2017; Elgin, 2007), have all led philosophers to conclude that the terminus of scientific inquiry need not be taken to be possession of true beliefs. Avoiding requiring adherence to factive norms in the midst of inquiry thus looks even more attractive when one considers that it may not even be the desired end state.

With one more assumption we think the arguments just reviewed also rule out justification norms as appropriate for governing individual contributions to collective inquiry. Our additional assumption is that whatever notion of justification is at play here it meets the total evidence requirement (Good, 1967). Our total evidence includes the information just provided about what kind of process science is. Not just how generally reliable our inquiry is, but also how reliable it is for hard problems in particular, and how reliable we are ourselves as individual or communal epistemic agents. For any one paper, the reasons one could produce in favour of its central claim could well be outweighed by these competing second-order considerations. Hence if one is to communicate the results of sustained inquiry on hard problems then one cannot limit one’s avowals to those a total-evidence respecting epistemic norm would permit. In the X-ray case, Bragg knew that the total evidence, on balance, did not support his corpuscular theory, but he nonetheless published and defended it publicly. This was not condemned by his contemporaries, rather they took his theory seriously despite everyone being aware that there exists compelling research which did not support it. So the surveyed justification norms of assertion cannot govern scientific avowals if they are to foster and permit publishing surprising findings or results, and we believe would not appropriately do so for any collective inquiry into a difficult or obscure matter.

Finally, one might think that none the less the belief norms may apply to public avowals. For all that has just been said, scientists may believe their claims to be true or justified, as long as scientists are ignorant of this history or these theoretical results. Or, perhaps, as long as they retain their faith in their own particular claims even while being aware that in general studies like theirs are not reliable. So long as scientists can maintain belief in themselves, we could require they only avow when they believe all they have said. Indeed, the International Committee of Medical Journal Editors might be requiring this when it says “[a]ll members of the group named as authors… should have full confidence in the accuracy and integrity of the work of other group authors” (ICMJE, 2013: 3).

We think such a retreat to mere belief or second order belief would be a mistake. For one thing, there is a large and persuasive literature in the philosophy of science detailing situations wherein inquiry may proceed well without scientists believing their claims (e.g. Kapitan, 1992; Dawes, 2013; Cabrera, 2018; Dellsén, 2018; Palmira, 2020, Fleisher, 2020). But over and above this we do not think that bad faith can be required of people. Quite generally we do not think a good social system can require participants to be ignorant as to the nature of their activities and their history, or require that they reason irrationally upon being informed about these things. False ideology or absurd arrogance should not be a prerequisite for inquiry. In this context it means that we cannot require as a condition of successful public avowal in science that scientists may not learn the various historical or theoretical facts that would undermine their faith in their assertions. Scientists do not need to believe that they are epistemically special in order to successfully participate in science.

If one agrees that bad faith cannot be mandatory then one can move from the above arguments to a rejection of both the justification norms and the belief norms. Scientists may well know of themselves that they are engaged in an activity which is not reliable for the kind of results they report. They thus may well not think their investigations are sufficient reason to believe their conclusions, or would suffice for justification all things considered. Their total evidence (inclusive of second order evidence) could not justify this, and they form their credences appropriately. Hence they may not believe their results at all, or believe them to be justified, and yet still properly avow. We ought not rule out as inapt these avowals of scientists who take the full measure of their epistemic situation. Hence building on the social epistemology of dividing cognitive labour, the pessimistic meta-induction and related theoretical results, we have been able to generalise our case study. This ruled out the factive, justification, and belief norms as the proper means of deciding what sort of conclusions are appropriately put forward as individual contributions to collective inquiry.

In some sense, of course, one could avoid this by just making weaker claims. Rather than saying X causes Y, one could say that according to one’s data X causes Y. The weaker claim might well be true, or at least justified even in light of total evidence, and perhaps ought to be believed in any case. Certainly it is the case that many scientific result claims are made in a hedged way in something like this fashion. Of course, at present we do not think that scientists always obey this norm of making only such weaker statements. However, if one was a strong advocate of one of the norms surveyed one could insist that strictly speaking only such weaker avowals would be proper, and fault those scientists who do not live up to this.

We note this possibility simply to note that it is a path we shall not explore here. We are concerned here with what should be expected of individual contributions to a broader inquiry, not really the exact forms they take. We are only making an analogy to the norms of assertion literature, not trying to weigh in on speech act theory. In the context of scientific research, we cannot only concern ourselves with events in a particular laboratory, or evidential relationships between particular data sets and a proposition. We also must put forward candidates for scientific communal uptake and potential targets for future inquiry. This goes all the more when one considers, as we have largely set aside, that one wishes ultimately to make use of scientific claims as the basis of public policy, where their external validity is what is of paramount importance (c.f. Cartwright, 2012 §4).

We do not doubt that it would be possible to reform scientific communication behaviour such that one sticks to scientific public avowals that are proper according to one of the surveyed norms by insisting on appropriate hedging. One could then just say explicitly (perhaps in so many words): “we also suggest such and such as a candidate for further investigation.” Or one could understand it to be an implicature that if Lab 1 reports that its inquiry suggested that X causes Y, Lab 2 might profitably investigate whether its own inquiry would suggest as much also. But our concern is not really with the precise linguistic form such claims would take so much as the social uptake amongst scientists. However results are conveyed, scientists must decide what claims are worthy of further tests (Friedman, 2020; Thorstad, 2021). Our point is that it would be inappropriate for scientists to insist that (in the absence of fraud or mistake or misfortune) these pursuit worthy claims must be true, or justified, or believed to be as much by their proponents. Further, we shall briefly outline a proposal for an actual norm that could appropriately govern, and is perhaps governing, scientific avowals in the next section, based on an analysis of the social epistemology of science. If one understands what norms avowals must satisfy in the good case, one can better adjust one’s attitude to them, without needing scientists to change their manner of communication.

## Primacy of the social

The upshot of the above is that scientific public avowals can, and in some cases ought, be allowed to be proper even when they are false, not justified in light of our total evidence, and neither believed to be true nor justified. While we are most concerned to show that this is normatively appropriate, we think this both matches the actual practice and standards of science and facilitates the enterprise of collective inquiry perpetuating itself successfully. The rejected norms were picked as the basis of our inquiry since they had been found plausible or defensible as norms for assertion, which is at least a somewhat related activity of putting forward scientific public avowals. So why this discrepancy? In short, we think this is because the social enterprise of inquiry requires that we allow people to be more lax in certain contexts than we normally require of individuals offering testimony, and through a long process of cultural evolution the scientific community developed norms of avowal to accommodate that fact.

It may seem surprising that the norms in science are less, rather than more, strict than everyday life. Intuitively science might seem to be a place where we operate under especially strict epistemic standards. And in a sense this is true, so long as one understands the "we" in a genuinely plural sense. That is to say, it is the group that must achieve high standards of rigour, and how individuals contribute to that may be somewhat indirect. For the group to achieve this goal we may well need the individuals who make up the group to be emboldened to take creative leaps and offer bold conjectures on matters that are complex, abstruse, and generally difficult to gain any epistemic purchase on. In this way our study of the somewhat niche topic of norms for avowals connects up with classic themes in the philosophy of science (e.g. Feyerabend, 1993; Popper, 2014), wherein it is emphasised that for science as a whole to progress individual scientists may operate in a somewhat gung-ho or ad-hoc manner.

We think that what is required here is some form of contextualist epistemic norm (see DeRose, 2002 for a factive version of this purported norm). Context, provided by the history and present consensus in a field, specifies some amount of the previous literature one must survey to check for coherence, and which methodological procedures one must carry out to reach a conclusion that is worth reporting to others. One’s avowal must be such that if one’s total evidence were what one had gathered in the methodologically proper way for one’s latest study, combined with whatever one has taken from the mandated subset of the previous literature contains, then one would be justified in believing one’s scientific public avowal. Public avowals are or should be held to specific subfield-specific norms thus generated—these norms for specifying the requisite literature to survey and adopting methods whose results are worth reporting on are implicitly taught to young scientists as part of their graduate and postdoctoral training.

Such a norm captures the sense in which researchers are held to demanding standards while remaining consistent with the various arguments we have offered showing that apt scientific public avowals need be neither true, known, believed, justified, reasonable to be believed, nor believed to be any such. We note that in the case of philosophy people have also argued that it is permissible to put forward claims even which one does not believe and are not justified (Barnett, 2019; Goldberg, 2013; Plakias, 2019). Hence if something like this norm is found to be operative in scientific fields it may well be a source of similarity between scientific and humanistic research. Coming to self-conscious understanding of such a norm may thus contribute in some small way to bridging the infamous two cultures divide.

The project for future inquiry would be to try and specify the details of what is implicitly being taught as normatively correct for putting forward avowals, and submit it to epistemic appraisal. To some extent, while not done explicitly under this aegis, the response to the replication crisis has led to just this discussion. Renewed consideration of standards of statistical significance and the ways in which journals decide what ought to be published have taken centre stage in scientists' discussions of their own practice. Rendering this more systematic and linking it more explicitly to epistemic theory would not only be interesting itself, but would also speak to the questions of science communication raised earlier. If we are to rely on scientific papers to guide policy and public discussion, it would be useful for us all to be clear on exactly what the epistemic status of the main claims of published scientific papers are. It would be inappropriate for us to rely on inter-scientific testimony as if they were put forward as proper assertions. Rather, we should read them as statements being put forward under very specific social epistemic contexts.

## Conclusion

When stating their central claims scientists should not be held to the kind of norms we hold assertions to if collective inquiry is to flourish. At the least, properly put forward scientific public avowals frequently do not and need not satisfy those norms of assertion that have been discussed in the analytic epistemology literature. Public avowals in science ought to be governed by a different norm.

We have suggested a contextualist justificatory norm as our proposal. But of course there are other possibilities, and this should be considered an area open for future inquiry. We note one especially interesting idea, drawing on the work of Yablo (2014). A long tradition sees science as in the business of putting forward partial truths, and our case study could be read that way. One could thus develop an account of the appropriate standards for scientific public avowals based upon a partial factive norm. It would take us very far afield to develop the technical machinery necessary to properly study this, and in any case, we prefer our contextualist justificatory account. However, we raise the possibility here partly to acknowledge it, and partly to make it apparent how much room there is for fruitful development in the theoretical study of what sort of norms should govern particular acts of putting forward conclusions in light of the wider project of collective inquiry.

Underlying all our arguments is the conviction that a scientific research community must ensure its members must spread out across logical space. We must allow for the exploration of different theories, by different methods, and accept that there will be different positions adopted as time goes by and results accumulate. Perhaps inquiry shall prove to be a process of never ending adjustment, and this will be our state in perpetuity. Or perhaps we may eventually learn from science what is actual. But even if so, in order to get there, we must allow that in the midst of inquiry, scientific public avowals will frequently be defences of implausible possibilities.
